# The NIST Primary Radon-222 Measurement System

**DOI:** 10.6028/jres.095.018

**Published:** 1990

**Authors:** R. Collé, J. M. R. Hutchinson, M. P. Unterweger

**Affiliations:** National Institute of Standards and Technology, Gaithersburg, MD 20899

**Keywords:** calibration, ionization chambers, measurement, radium-225, radon-222, standards

## Abstract

Within the United States, the national standard for radon measurements is embodied in a primary radon measurement system that has been maintained for over 50 years to accurately measure radon (^222^Rn) against international and national radium (^226^Ra) standards. In turn, all of the radon measurements made at the National Institute of Standards and Technology (NIST) and the radon transfer calibration standards and calibration services provided by NIST are directly related to this national radon standard. This primary radon measurement system consists of pulse ionization chambers and ancillary gas handling and gas purification equipment. The system is currently undergoing a significant upgrading and expansion which will replace the extant outdated system.

## 1. Introduction

Unlike the calibration for many other radionuclides, there is no currently available or demonstrated procedure for fundamentally or directly calibrating radon (^222^Rn) without recourse to an indirect or comparative measurement. As a result, at the present time, all measurements of radon are ultimately related back to some radium (^226^Ra) standard. Within the United States, the National Institute of Standards and Technology (NIST), formerly the National Bureau of Standards (NBS), maintains both national and international radium standards that can be directly related to the international primary mass standards of radium prepared by Marie Curie in 1911 and Otto Honigschmid in 1934 [1].

A primary measurement and calibration system for ^226^Ra and ^222^Rn has been maintained by NIST/NBS since the early 1940s [2]. This system was and still is one of the principal tools used to compare and trace radium standards to each other, as well as to relate radon measurements to radium standards.

The system consists of pulse ionization chambers which are used in conjunction with radium solution standards and ancillary gas handling and gas purification equipment. Use of the system relies upon a methodology that was originally developed for the purpose of assaying and standardizing radium solutions, such as comparing various preparations with each other or with national or international radium standards [2,3,4].

In this method, often referred to as the “radon method of analysis of radium,” the radon generated from the decay of radium in a sample or standard is physically separated from the radium, and quantitatively transferred to the ionization chambers. Alpha particles resulting from the decay of the radon and the radon decay products are then detected and counted in the chambers. In addition to using the method for comparing laboratory-prepared solutions and standards of radium, laboratories here employed it for radium assay of a variety of samples (e.g., water and wastewater samples and various solutions of solid samples such as soils or uranium ores) [2,3,4]. Similarly, the method also has been used to measure the radon content of whole air samples. Such measurements were extensively performed by this laboratory in the late 1940s to assay mine atmospheres and breath samples from uranium-mine workers and radium-dial painters [2].

Inasmuch as this measurement system and methodology provides a quantitative link between a radon measurement and the radium content of a sample or standard, it also has served and continues to serve as a national calibration standard for radon measurements. In fact, this national standard, embodied in the primary measurement system, has been maintained for over the past 50 years for this purpose of accurately measuring radon against international and national radium standards. In turn, all radon measurements made by NIST and the radon transfer calibration standards and calibration services provided by NIST are directly relatable to this national radon standard [5,6].

## 2. Requirements for a Primary Calibration Standard

Before directly addressing the topic of the NIST primary radon measurement system and its application to other NIST radon transfer standards and calibration services, it may be useful and insightful to summarize what we believe are the necessary requirements for a primary calibration standard. The requirements are that the standard should: (i) have long-term stability; (ii) be sufficiently accurate to meet the calibration and subsequent measurement needs; and (iii) be efficacious for cross calibration purposes, allowing the transfer of calibrations to other measuring instruments and methods.

One of the great advantages of a calibration system based on ionization chambers over other measurement instruments (e.g., scintillation cells) is its ability to maintain a constant and stable calibration over very long time periods. Calibration results from this laboratory, as well as from the U.S. Department of Energy Environmental Measurements Laboratory [7], have demonstrated that radon calibration factors for ionization chambers have remained constant within a few percent for decades. This level of stability provides a useful independent check on the joint consistency of both the use of a radium standard and the instrument performance. With other instruments such as scintillation cells which are easily subject to physical degradation and subsequent changes in detection efficiency, one must assume, without verification, that the radium standard has remained constant and that the use of the standard has not changed.

In regard to the second requirement, overall calibration accuracies in the range of 1 to 2 percent are possible with ionization-chamber-based calibration systems. This appears to be more than adequate for presently envisaged radon measurement needs (see the workshop discussion in the Preface to this issue).

The third requirement is that the primary calibration standard must be capable of handling samples in a way that allows for a sufficiently accurate cross calibration to secondary calibration systems. The ancillary gas-handling and gas-purification manifold incorporated into the NIST primary system is very efficacious for transferring identical radon-in-air samples measured with one system to the ionization chambers for a direct calibration. The manifold also can be used to prepare replicate calibrated samples from radium sources.

## 3. The Pulse-Ionization-Chamber-Based Primary System

**Figure 1:**
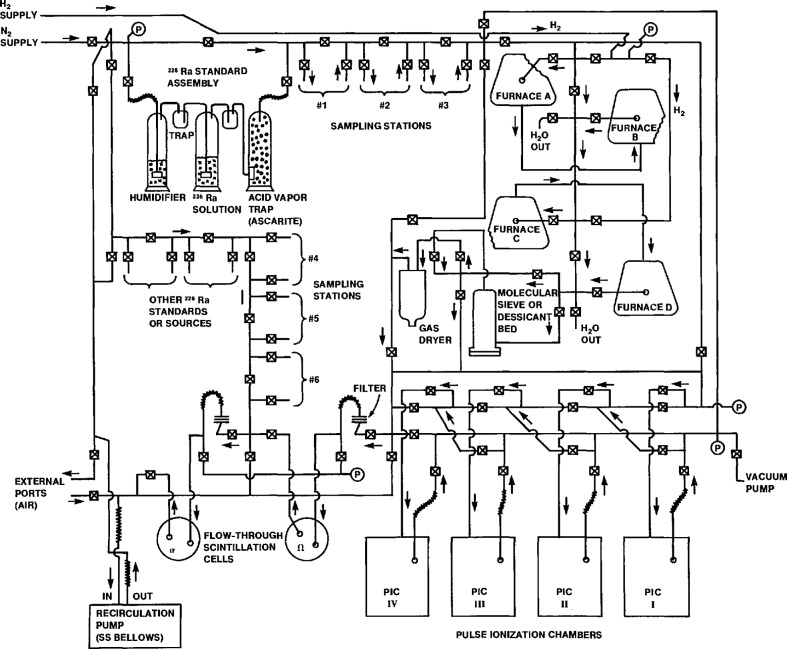
A simplified schematic of the NIST primary radon measurement system illustrating its major components and principle of operation.

The NIST primary radon measurement system comprises three major components: (i) a manifold containing a radium standard source and a sampling location for processing gas sample bulbs; (ii) gas purification equipment; and (iii) the ionization chamber and appropriate electronics. A simplified schematic illustrating these components and the basis of their operation is shown in figure 1.

**Figure 2:**
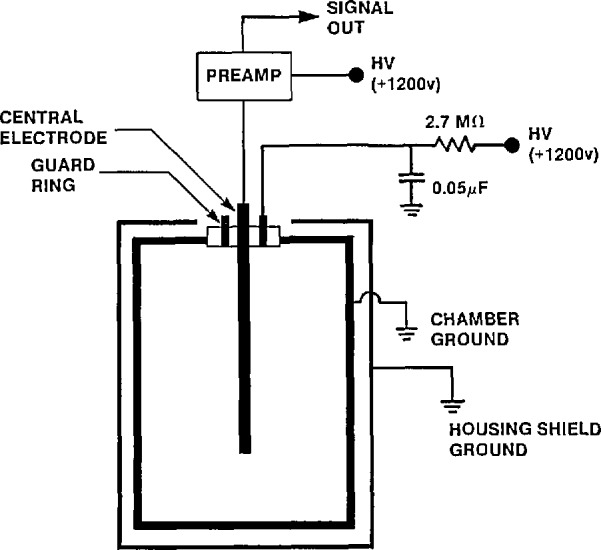
A block diagram of a pulse ionization chamber showing its electrical connections for operating in a pulse counting mode.

The system employs an internal gas counting technique in which a radon sample is introduced, together with a suitable filling gas, directly into a chamber. The chambers consist of a nominal 4-L volume cylindrical cathode with a central wire anode. Their operating voltage is maintained in the ionization region (typically 1200 V); and they are operated in a pulse counting mode (see fig. 2). The advantages of counting current pulses over a continuous direct current mode of operation has been described previously [3].

The principles of operation are straightforward. Alpha particles resulting from the decay of radon and its decay progeny ionize the gas contained in the chamber. Electrons and positive ions liberated in the ionized gas are collected by means of the electric field maintained by the high voltage between the central electrode and chamber wall. The collection of the electrons on the central electrode produces a pulse of current to flow in an output resistor R. Since the voltage drop across R is proportional to the current flowing, the voltage resulting from the passage of a single alpha particle will increase and then decrease again as the current pulse fades away. This voltage pulse is amplified and then recorded by an electronic counting circuit.

Pure nitrogen is used as the filling gas since it has a very low electron affinity which allows for a sufficiently sharp and distinct pulse with a fast collection time of the electrons on the electrodes. Resolving times of less than 100 μs are readily achievable [3]. The performance of the chambers is very adversely affected by the presence of ions and condensable vapors in the gas sample, as well as by the presence of electronegative gases (such as oxygen). Therefore, an ancillary gas-handling and gas-purification capability is necessary.

The gas-handling manifold is used for processing samples i.e., separating and purifying the radon from whole air samples, mixing it with the filling gas, and transferring the gas into counting chambers that have been previously evacuated. This type of radon gas-handling system, commonly associated with methods for assaying radium samples, has been developed for many applications by Evans [8], by Curtiss and Davis [3], and by Harding, et al. [4]. Similar systems also have been described by Blanchard [9], by Harley [10], and by Kreiger and Jacobs [11].

With the system illustrated in figure 1, radon samples (either connected at the sampling location or obtained by generation from the radium standard) are transferred with a slow stream of nitrogen carrier gas through the purification equipment that removes water vapor and oxygen from the gas stream. Oxidative purification is achieved with a quartz combustion tube that is filled with tightly wound copper gauze and surrounded by a furnace operated at 500 °C. The heated copper in the combustion tubes is previously reduced by a stream of hydrogen before it is used to purify the samples. Samples transferred to the ionization chamber are counted after the radon and its short-lived decay products reach secular equilibrium.

The ionization chambers are calibrated against ^226^Ra standards (sec. 5.1) by quantitatively transferring known accumulated amounts of radon into the chambers. This transfer is performed by slowly bubbling the nitrogen carrier gas stream through a standardized radium solution which is contained in a modified gas-washing bottle. As in the procedure used for measuring radon samples, the gas stream passes through the purification system and fills the pre-evacuated chambers. Great care must be taken to insure that the transfer of radon from the radium solution to the chamber is 100% efficient, and that it replicates as closely as possible the conditions of the gas transfer for a radon sample [12].

The original NIST/NBS system, built in the 1940s, consisted of a bank of over 20 chambers. It was used for many years to perform routine measurements of air and breath samples—up to over 1200 samples per year throughout the late 1940s and 1950s. Although the system underwent many modifications over the intervening years (particularly changes in the gas-handling manifold, modernization in the electronics and signal processing, and taking some of the chambers out of service), some of the original chambers always have remained in continual use. These original chambers were constructed of silver-soldered brass with rather obsolete vacuum seals and electrical insulators, and were wax sealed to an all-glass gas-handling manifold. In recent years, it became increasingly apparent that this ancient system was rapidly becoming inoperable. Of the four remaining chambers, only two are usable, and both of these have leaks that are increasing in seriousness. The chamber backgrounds, because of the 40-year accumulation of deposited ^210^Pb, preclude measurements at typical environmental concentration levels. As a result of the system’s deteriorating condition and the reemphasized importance of this national standard, the design and construction of a replacement system was initiated in 1988.

## 4. The New Primary System

The new primary system is a modernized and expanded version that is based on the design of the original system. At the time this new system was being designed, consideration was given to possibly selecting an alternative measurement method for use as the primary standard. Despite the passage of 50 years, no other available method or instrumentation could be found that was more advantageous or superior to the original pulse ionization chamber.

**Figure 3:**
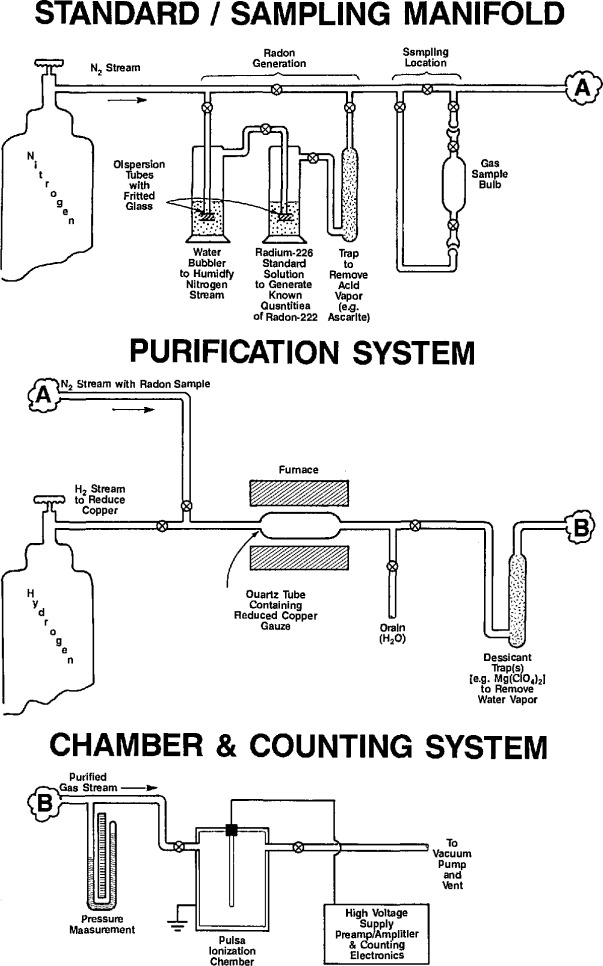
Schematic of the new NIST primary radon measurement system showing the complete gas-handling and gas-purification manifold used in conjunction with the pulse ionization chambers.

A schematic representation of the new system is shown in figure 3. Despite the seeming complexity of the extensive gas handling and purification manifold, its principle of operation is virtually the same as the simple system illustrated in figure 1. The new manifold incorporates a similar radium standard assembly, various sampling stations, a gas purification network based on similar heated copper combustion tubes, and the necessary traps, desiccant beds, pressure and flow rate measurement devices, gas supplies, and vacuum lines. As part of its increased versatility, it also incorporates a recirculation pump for possible experiments requiring recirculated flow, as well as two flow-through ZnS(Ag) scintillation cells which can be used for less exacting radon assay measurements. All of the piping and valves for the manifold are of stainless steel high-vacuum construction with the exception of the modified gas-washing bottles for the radium standard and the quartz combustion tubes which are connected via glass-to-metal transition tubes.

**Figure 4:**
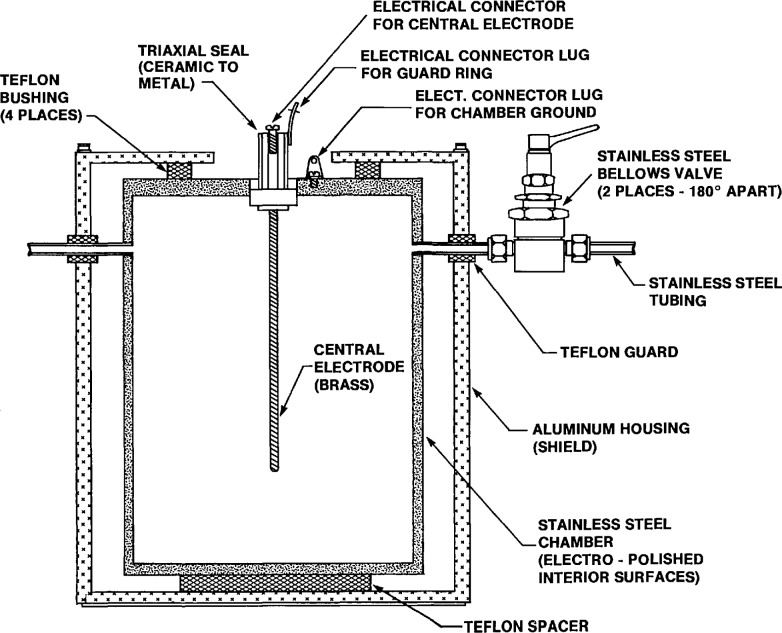
Cross section of the new NIST pulse ionization chamber.

For the system, four new custom-made ionization chambers were fabricated by a commercial vendor (Reuter-Stokes^1^) based on the original chamber design [3,4] and according to specifications provided by our laboratory. It was intended that these replacement chambers be of the same volume to obtain approximately the same detection efficiency and that they perform as nearly identically as possible as the originals, and that intercomparisons between the chambers be performed before the old ones are taken out of service. The new chambers, illustrated in figure 4, are fabricated out of stainless steel with completely sealed welded seams, unlike the originals which had O-ring gasket seals that were susceptible to leaks over time. All of the interior surfaces are electro-polished to minimize electrostatic field irregularities and to minimize surface deposition of the radon decay products. Electrical insulation of the central electrode and guard ring is made with the vendor’s proprietary triaxial ceramic-to-metal seal that was welded as a complete assembly to the chamber top. The volume of one of the original chambers was measured by filling with water and was found to be 4.248 L. Based on dimensional tolerance limits, the new chambers have a minimum volume of 4.234 L and a maximum volume of 4.261 L.

Electrical coupling of the chambers for power and signal processing is as shown in figure 2. The resolving time of the output signal pulses is approximately 10 μs. These pulses are amplified and pulse height analyzed in the system’s own dedicated computer.

**Figure 5:**
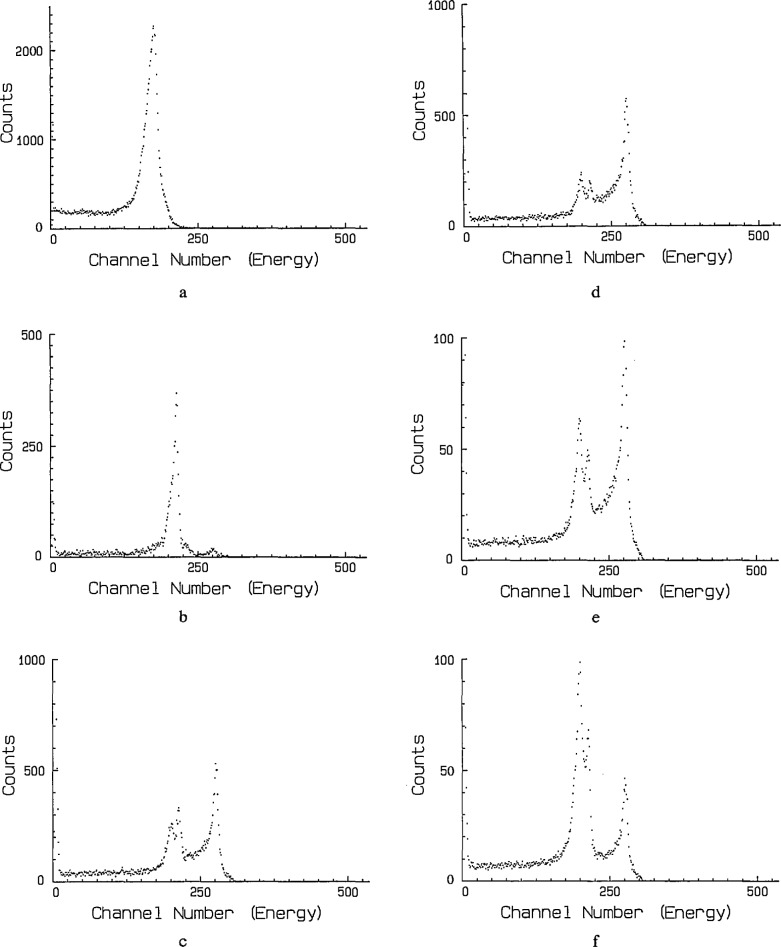
Typical spectra of ^222^Rn and its decay products obtained with a pulse ionization chamber, (a) 100-s count immediately after filling; (b) 100-s count immediately after flushing; (c) 1000-s count 10 min after flushing; (d) 1000-s count 35 min after flushing; (e) 3000-s count 60 min after flushing; (f) 5000-s count 150 min after flushing. See text for details.

Typical spectra obtained with the new chambers are shown in figure 5. They depict the alpha-particle spectrum shapes as they develop in time after the introduction of radon into the chamber, followed by the almost immediate removal of about 99% of the introduced radon. Figure 5a shows the 5.49 MeV peak of the monoenergetic spectrum of almost pure ^222^Rn before the 3-inin ^218^Po and subsequent progeny have had time to grow to significant proportions. Shortly thereafter, a significant amount of ^218^Po “grows in” from decay of the radon so that when most of the radon is flushed out of the chamber, the 6.00-MeV alpha particle peak of ^218^Po dominates the spectrum, as shown in figure 5b. A small ingrowth of ^214^Po is also evident as seen by the small peak at 7.69 MeV. The spectrum in figure 5c, taken 10 min after flushing, shows that the ^218^Po (6.00 MeV peak) has decayed to a level comparable to the residual radon (5.49 MeV peak). At the same time, the 7.69 Mev peak from ^214^Po has begun to more rapidly “grow in” at a rate genetically determined by the half-lives of the earlier members of the radon decay chain (26.8-m ^214^Pb and 19.9-m ^214^Bi). Figure 5d, obtained 35 minutes later, shows the ^218^Po in secular equilibrium with the radon and the continued growth of the ^214^Po. Although they are in equilibrium, note that the ^218^Po count rate is lower than that for ^222^Rn. Presumably this is because the detection efficiency for the gaseous radon is near 100 percent whereas the polonium, which attaches to the surfaces within the chamber, has a significant fraction of the emitted alpha particles absorbed by these surfaces without detection. Lastly, figures 5e and 5f show the gradual establishment of secular equilibrium between all members of the decay chain beginning with ^222^Rn when the ^222^Rn is not removed.

Calibrations with radium standards for the new chambers are not complete, but preliminary results indicate that the overall detection efficiency for radon in secular equilibrium with its decay products is very near the expected value of 2.00 counts per s per Bq of ^222^Rn. The four remaining old chambers had efficiencies which ranged from 1.96 to 2.01 cps Bq^−1^. The value of approximately 2 arises from the ^222^Rn decay being detected with an efficiency of approximately 100% while the ^218^Po and ^214^Po are detected with an efficiency of about 50%. Losses in efficiency and spectrum peak shifts are very noticeable with incomplete oxygen removal or the presence of other impurities in the chambers. Typical calibration reproducibilities in terms of a standard error of the mean are a few tenths of one percent for counting time intervals of 8 to 10 h at radon activity concentrations of about 0.5 to 1 Bq L^−1^. A substantial fraction of this uncertainty arises from the statistical “counting error” imprecision which could be further reduced and minimized with longer counting time intervals.

Background count rates for the new chambers, which primarily determine the measurement sensitivity, are approximately 0.008 counts per s. For a 4-L chamber volume, this corresponds to a background equivalent radon activity concentration of 0.001 Bq L^−1^ (or 0.03 pCi L^−1^).

The new system presently is undergoing extensive testing and evaluation. Many additional tests, such as for adsorption losses of radon in traps, for the degree of gas purification, and for the transfer efficiencies as a function of flow rate and pressure differences, will have to be completed before the system is fully evaluated and operable. Conceptually, an overall accuracy (more correctly an *in*accuracy) for this primary measurement system of plus or minus several percent is achievable. However, uncertainties of this magnitude will be achieved only if the gas samples are carefully and quantitatively processed and transferred to the chamber, and if the chamber is accurately calibrated. Inasmuch as the system relies on comparative measurements, i.e., the assay is performed by comparing the output from an unknown radon sample with that from a known radium standard, the uncertainty in the radium standard and in its use is of paramount importance. In addition, it is clear that additional major sources of inaccuracy arise from errors in the gas-handling procedures used during both the sample processing and in the chamber calibration. These gas-handling procedures and subsequent counting techniques are a very complicated sequence of many individual operations or steps. It must be recognized that virtually every step in this sequence introduces a conceivable source of inaccuracy. The earlier evaluations performed in characterizing the original system [12] may no longer be completely applicable, and some of the present procedures used with that system may not withstand complete critical scrutiny. Therefore, it is necessary that the new system undergo a detailed evaluation for each step in order to obtain a complete uncertainty assessment.

## 5. Application to Other NIST Radon Transfer Standards and Calibration Services

**Figure 6:**
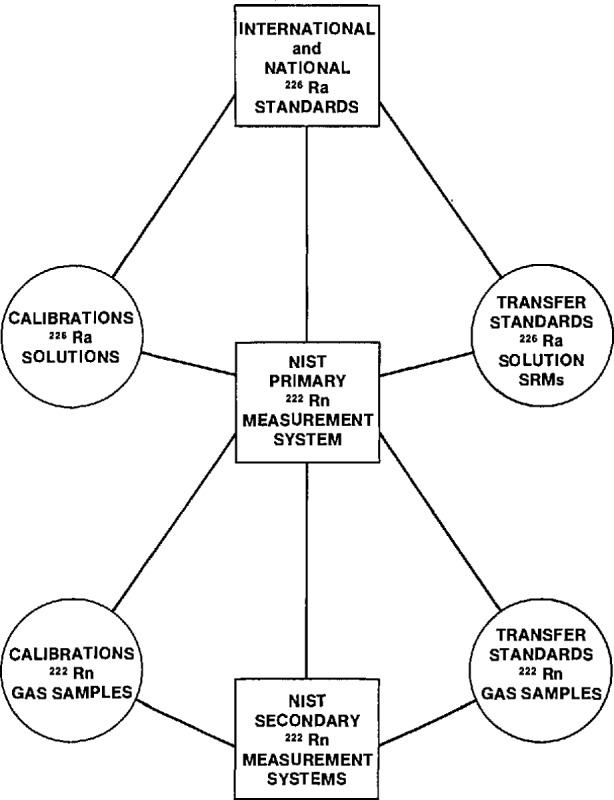
Illustration of the relationship of the NiST primary radon measurement system to other NIST radium and radon standards and calibrations.

As indicated, the pulse-ionization-chamber-based primary measurement system serves as the national standard for radon measurements and as such serves as an underpin to the entire NIST radon program. Its central role in linking all NIST radon and radium measurements, transfer standards, and calibration services is diagrammatically illustrated in figure 6. Brief descriptions of these transfer calibration standards and services, as well as some of the NIST activities and facilities used to support them are described below.

### 5.1 Radium-226 Standard Reference Materials

NIST ^226^Ra solution Standard Reference Materials (SRMs) are one of the principal transfer standards for radon measurements [5]. Various series of these standards have been available since the mid-1940s. The current series of three SRMs contain approximately 5 to 20 g of solution in a flame-sealed ampoule and have a radium content ranging from 10^−14^ to 10^−8^ g. A few standards in an additional series of ^226^Ra gamma-ray solution standards are also available. They contain 5 g of solution and have a radium content ranging from 10^−7^ to 10^−4^ g. These later standards can be used for radon analysis at higher concentrations.

Most laboratories maintaining an independent radon calibration capability use these radium transfer standards as their “primary” standard for radon. Although more capable laboratories can be expected to utilize this standard with success, it is not a very practical transfer standard for laboratories which do not maintain a large internal calibration capability. The use of these radium solution standards requires an indirect radon calibration based on a very careful quantitative extraction, transfer, and collection of radon from a solution.

### 5.2 Radon Calibrations and Measurement Intercomparisons

Radon calibrations for other laboratories can be performed at NIST by either assaying the radon concentration in a gas sample bulb which is sent by the laboratory, or by sending a sample bulb filled with a known radon concentration to the laboratory for their assay. In either case, the radon concentration in the assay measurement made at NIST or in the sample bulb filled at NIST is directly relatable to the primary radon measurement system.

Approximately 2 years ago, a new secondary measurement system for these calibrations was introduced at NIST. A NaI(Tl) well counter was cross calibrated against the primary system. This detector used in conjunction with spherical 25-mL glass ampoules containing radon samples was shown to be very reproducible and fairly independent of geometry effects. The method has a wide dynamic range and can be used for radon-in-air samples from the picocurie level to 100 nanocurie level.

This system was used for a recent measurement intercomparison of five principal laboratories within the United States that maintain an independent radon calibration capability based on radium solution standards. An unsatisfactorily large, systematic measurement discrepancy of over 7 percent among these laboratories was found despite the fact that all of the laboratories’ calibrations were based on similar NIST radium solution standards. Our laboratory is continuing to work with these other laboratories to try to understand and resolve this discrepancy.

An international radon measurement intercomparison among international national metrology laboratories is also being planned for 1990.

### 5.3 Radon-in-Water Standard Generator

In 1986, NIST completed the development of a transfer standard for radon-in-water measurements. This work was initiated at the request of the U.S. Environmental Protection Agency to support the extensive national survey of radon concentration in drinking water supplies.

This standard consists of a polyethylene-encapsulated radium solution source in a small-volume accumulation chamber and an ancillary mixing and dispensing system. It generates aqueous solutions of radium-free radon of which multiple aliquots may be dispensed and used as standardized solutions for calibrating radon-in-water assay procedures. As in all other NIST transfer standards for radon, the generator calibration is directly relatable to the primary radon measurement system. The overall uncertainty in the radon calibration for a sample aliquot dispensed from the generator (such as into liquid scintillation vials) is estimated to be approximately 4%.

Additional details on the development, operation, and performance of the standard generator may be found in Hutchinson, et al. [13,14].

### 5.4 Radon Flux Density Standard

A project to develop a large surface area flux density standard (i.e., the radon exhalation rate per unit area) has been underway for several years [15].

A few attempts have been made by other laboratories to obtain a radon flux density calibration facility based on the characterization of a fabricated site of either natural or enriched radiumbearing soils, sands, or mill tailings. The novel, alternative approach considered for the NIST standard consists of incorporating an aqueous solution of radium into a sealed, shallow, large-surface-area container which is covered with a thin, but rigid, polyethylene sheet which is permeable to radon. The use of a radium solution eliminates possible spatial dependencies and minimizes the effects of ambient and meteorological variations. This source configuration is expected to exhibit purely diffusive (Pick’s Law) radon transport properties without a permeation (Darcy’s Law) transport component which is one of the troublesome and unpredictable aspects of radon movement in solid, porous media. A steady-state diffusion model for such a source configuration has been derived by Rubin [16] as an extension of the various radon transport cases treated in Collé et al. [17].

A 40-cm diameter prototype has been operational for the past 3 years. Preliminary evaluations for the constrained and unconstrained flux density have been completed. Although the efficacy of the flow-dependent extrapolation procedures used for calibrating the source were verified, problems arising from transpiration and evaporation of water through the polyethylene surface precludes the development of the prototype in its present configuration. Alternative designs incorporating small encapsulated radium sources under a rigid metal screen are under consideration.

### 5.5 Radon Transfer Standards Based on Polyethylene-Encapsulated Radium Sources

Based on our studies for the radon-in-water standard generator and the flux density standard prototype (sees. 5.3 and 5.4), it is believed that small polyethylene-encapsulated radium solutions would act as purely diffusive sources of radon that could be successfully used as transfer standards in a variety of configurations and measurement applications. The efficacy of such sources has been demonstrated in the development of the radon-in-water standard generator. These capsules would be an alternative or replacement for the present radium solution SRMs (see sec. 5.1).

Studies for their production and calibration have recently been initiated. At the present time, it is envisaged that the sources may be encapsulated using techniques originally developed for trace gas “permeation” tube calibration standards [18]. The development of suitable protocols for their use as SRMs also would be performed.

### 5.6 A Primary Calibration Based on Liquid Scintillation Counting

NIST also has work underway on a new initiative to perform a primary radon calibration which is not based on an indirect or comparative measurement against a ^226^Ra standard. This may serve not only as an alternative primary radon calibration, but also as an independent verification of the primary radium standards. This calibration, expected to have an overall uncertainty of 1 to 2 percent, is based on liquid-scintillation-counting techniques using radium-free radon solutions obtained from the radon-in-water standard generator (see sec. 5.3). To date, extensive liquid scintillation measurements of radon-in-water samples have been made as a function of total sample volume and cocktail/water ratio. Comparative measurements against similarly prepared tritium (^3^H) water samples have been initiated to fix a parameter in the model used to calculate the efficiency for counting radon progeny decaying by beta-particle emission. This primary calibration, if successful, will be a significant achievement in providing the first independent confirmation of the radium-based calibrations used over the past 50 years.

## Footnotes

1 Certain commercial equipment, instruments, or materials are identified in this paper to specify adequately the experimental procedure. Such identification does not imply recommendation or endorsement by the National Institute of Standards and Technology, nor does it imply that the materials or equipment identified are necessarily the best available for the purpose.
